# Hyperbaric Oxygen Therapy Promotes Hearing Gain with Increases in Serum IGF-1 and HSP70 in Patients with Idiopathic Sudden Sensorineural Hearing Loss

**DOI:** 10.1155/2022/1368783

**Published:** 2022-10-25

**Authors:** Yi Zhang, Xingyuan Jia, Xuehua Liu, Lin Zhao, Yan Zhou, Fang Liang, Yu Gao, Jing Yang

**Affiliations:** ^1^Department of Hyperbaric Oxygen Medicine, Beijing Chaoyang Hospital, Capital Medical University, Beijing 100020, China; ^2^Institute of Basic Medicine Sciences, Beijing Chaoyang Hospital, Capital Medical University, Beijing 100020, China; ^3^Department of Hyperbaric Oxygen Medicine, Beijing Pinggu Hospital, Beijing 101200, China

## Abstract

**Objective:**

Hyperbaric oxygen therapy (HBOT) has been recommended for the initial and salvage treatment of patients with idiopathic sudden sensorineural hearing loss (ISSHL), but its underlying mechanisms remain unclear. In this study, we investigated whether HBOT alters serum levels of insulin-like growth factor 1 (IGF-1) and heat shock protein 70 (HSP70) in patients with ISSHL. Then, we identified the relationship between hearing recovery and changes in serum IGF-1 and HSP70 levels.

**Methods:**

Moderately severe to profound unilateral ISSHL patients (*n* = 70) and healthy control participants (*n* = 30) were enrolled. The ISSHL patients were randomly assigned to receive medical therapy alone (MT group, *n* = 35) or both HBOT and medical therapy (HBOT + MT group, *n* = 35). Audiometric testing was performed before and after treatment. Serum IGF-1 and HSP70 levels were assessed by ELISA in ISSHL patients pre-and posttreatment and healthy controls.

**Results:**

Before treatment, compared with the healthy controls, serum IGF-1 and HSP70 were lower in ISSHL patients. After treatment, serum IGF-1 and HSP70 increased in both the HBOT + MT and MT groups, although they were significantly higher in the HBOT + MT group (*p* < 0.01). In the HBOT + MT group, these increases were associated with hearing gains. In addition, IGF-1 was strongly associated with HSP70 (*r* = 0.621, *p* = 0.001). No such association was found in the MT group (*p* = 0.757).

**Conclusion:**

Administering HBOT in addition to medical therapy can improve the hearing of patients with moderately severe to profound unilateral ISSHL. The improvement is related to the upregulation of IGF-1 and HSP70.

## 1. Introduction

Acute hearing loss adversely affects quality of life; most cases are sensorineural. Sudden loss of sensorineural hearing (which is idiopathic in 90% of cases) is defined as a rapid hearing loss of ≥30 dB in ≥3 contiguous frequencies within ≤72 h [1]. It affects approximately 5−27 per 100,000 people annually, but its incidence has been increasing over the past decades [1–3]. Although its pathogenesis is incompletely understood, the most common triggers involve viral infections, vascular disorders, and autoimmune factors [1, 4].

Functional impairment/loss of cochlear hair cells (HCs) is the most common cause of sensorineural hearing loss, so protection and regeneration of HCs are essential for treatment [5]. There is evidence that insulin-like growth factor 1 (IGF-1) and heat shock protein 70 (HSP70) are associated with HCs survival. IGF-1, a vital growth mediator, acts on supporting cells to maintain the number of HCs; exogenous IGF-1 has been reported to promote the regeneration of cochlear afferent synapses. [5, 6]. In adult mammals, IGF-1 is primarily produced by the liver, secreted into the plasma, and reaches the inner ear via blood [7]. IGF-1 deficiency may be involved in the pathophysiology of sensorineural hearing loss in Turner syndrome [8]. A cohort study showed that higher serum levels of IGF-1 among individuals aged 50 to 60 years were associated with a decreased risk of hearing loss during a six-yearfollow-up [9]. However, the characteristics of serum IGF-1 in patients with idiopathic sudden sensorineural hearing loss (ISSHL) remain unclear. Like IGF-1, HSP70 also protects HCs by acting on supporting cells [10]. HSP70 is a stress-inducible heat shock protein that protects HCs from aminoglycosides and cisplatin [11, 12]. In 2006, Park et al. [13] found that serum levels of HSP70 are significantly higher in patients with ISSHL.

Increased oxygen delivery to the inner ear may aid hearing recovery due to the high oxygen demands and relatively limited vascular supply of the cochlea [4]. Hyperbaric oxygen therapy (HBOT) is a therapeutic approach in which the patient breathes 100% oxygen while being exposed to an ambient pressure of >1 atmosphere absolute [14]. Studies have shown that the oxygen tension in the intracochlear perilymph increases significantly during HBOT [15]. Clinically, HBOT is used both as an initial therapy and as a rescue therapy for ISSHL [1]. However, its underlying molecular mechanisms remain unclear. Specifically, it is not known whether HBOT changes the serum IGF-1 and HSP70 levels in patients with ISSHL.

In this study, we found that HBOT changed serum IGF-1 and HSP70 levels in ISSHL patients and explored the molecular mechanisms underlying the HBOT-induced hearing improvements in ISSHL.

## 2. Patients and Methods

### 2.1. Participants

Between January 2017 and December 2019, we enrolled 70 ISSHL patients with moderately severe to profound hearing loss from Beijing Chaoyang Hospital, Capital Medical University. The inclusion criteria were unilateral ISSHL of ≤7 days duration and hearing loss of ≥56 dB (evaluated by the pure-tone average, PTA). The diagnosis was made by the otolaryngology department. The exclusion criteria were as follows: (i) mild to moderate hearing loss (26–55 dB); (ii) bilateral ISSHL; (iii) Meniere's disease, cholesteatoma, or otitis media; (iv) previous otologic surgery; (v) anatomic abnormality of the inner ear and/or auditory pathway; (vi) previous systemic therapy with an ototoxic drug affecting the central nervous system; (vii) uncontrolled diabetes mellitus (glycated hemoglobin >10%); (viii) contraindication to HBOT (e.g., poor function in the eustachian tube, pneumothorax, or claustrophobia); and (ix) unwillingness to undergo HBOT.

Using a random number table, the 70 enrolled ISSHL participants were randomly divided into two subgroups (*n* = 35 per group): an HBOT and medical therapy (HBOT + MT) group and a medical therapy (MT) group. Thirty age-matched healthy individuals without hearing loss, ear disease, or other systemic diseases were recruited as the healthy control group. The protocol was approved by the ethics committee of Beijing Chaoyang Hospital, Capital Medical University (2016-88). Written informed consent was obtained from each individual before participation in the study.

### 2.2. Treatment

All ISSHL participants were treated with the same medical therapy protocol based on Chinese guidelines [16]. For corticosteroid therapy, patients received prednisone (1 mg/kg body weight per day; maximum 60 mg) for 5–6 days, followed by a tapering dose over the subsequent 5 days. For adjuvant therapy, 70 mg of ginaton (*Ginkgo biloba*) (Ji Sheng Chemical Pharmaceutical Co. Ltd, Taiwan, China) was intravenously administered for 14 days, plus 3 × 0.5 mg oral methylcobalamin (Eisai China Pharmaceutical Co., Ltd, Suzhou, China) for 20 days.

HBOT was initiated one day after enrolment; 100% oxygen was administered at a pressure of 2.0 atm during 1 h daily sessions for 20 days (days 1 to 20).

### 2.3. Audiological Evaluation

Pure-tone audiometry was carried out on the day of enrolment (Day 0) and one day after the last session of HBO treatment (or Day 21 for the medicine group) to obtain hearing levels at 250, 500, 1000, 2000, 4000, and 8000 Hz. The PTA was calculated as the average value at 500, 1000, 2000, and 4000 Hz. The severity of hearing loss was classified as moderately severe (56–70 dB HL), severe (71–90 dB HL), or profound (≥91 dB HL).

The outcome was measured according to Heuschkel1 et al. [17] as absolute hearing gain (ΔPTA) = (initial PTA) − (post-therapy PTA) in dB. A ΔPTA <15 dB was considered to indicate that no recovery had occurred [16].

### 2.4. Laboratory Evaluations

Blood samples were drawn from all ISSHL participants from the median cubital vein upon enrolment, and on Day 21, samples were centrifuged to obtain the serum fraction which was stored at −80°C until analysis. Serum levels of IGF-1 and HSP70 were determined using a Human IGF-1 ELISA kit and a Human HSP70 ELISA kit (R&D Systems, Minneapolis, MN, USA), respectively, according to the manufacturer's protocols. The change of IGF-1(△IGF-1)=(post-therapy IGF-1) − (initial IGF-1) and the change of HSP70(△HSP70) = (post-therapy HSP70) − (initial HSP70).

### 2.5. Statistical Analyses

Statistical analyses were carried out using IBM SPSS 26.0 (Armonk, NY, USA). Continuous data are shown as the mean ± standard deviation. The Shapiro–Wilk test was used to assess data normality. A one-way analysis of variance or the Kruskal–Wallis H test was used to compare healthy controls and the two ISSHL groups, Student's *t*-tests or the Mann–Whitney U nonparametric tests were used to compare the duration, PTA, and changes of IGF-1 and HSP70 between the two ISSHL groups. Paired *t*-tests or the Wilcoxon signed-rank sum test were used to compare the same patient before and after treatment. Fisher's exact test was used to test categorical data. Pearson correlation test or nonparametric Spearman correlations tests were used to analyze the relationships between parameters among the groups. *p* < 0.05 was considered to indicate significance. All statistical tests were two-sided.

## 3. Results

### 3.1. Clinical Characteristics

The clinical characteristics of the study cohort are shown in Table 1. There were no significant differences in sex, age, affected ear, the interval between onset of hearing loss and initial treatment, degree of hearing loss, comorbidities, or accompanying symptoms that could affect hearing outcomes between the MT and HBOT + MT groups (*p* > 0.05). There were no significant differences in age or sex between the two ISSHL groups and the healthy control group (*p* > 0.05).

### 3.2. Clinical Outcomes

The recovery rate was markedly higher in the HBOT + MT group (80.0%) compared to the MT group (57.1%, *p*=0.035).  Table 2 shows the improvement of hearing thresholds in the two ISSHL groups. There was no significant difference in initial hearing between the two groups. Following the respective treatments, hearing improved in both the groups (*p* ≤ 0.001). Following treatment, the PTA in the HBOT + MT group was significantly lower than in the MT group (*p*=0.019). The HBOT + MT group experienced significantly higher hearing gains than the MT group (*p* ≤ 0.001).

### 3.3. Serum IGF-1 and HSP70 Levels in Patients with Various Severities of ISSHL

**Figure 1:**
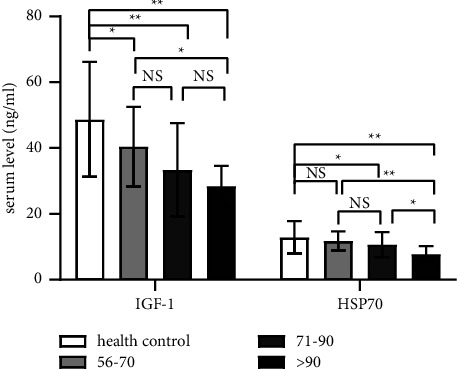
The characteristics of serum IGF-1 and HSP70 levels in patients with various severities of ISSHL. ^∗^*p* < 0.05. ^∗∗^*p* < 0.01. ns: *p* > 0.05.

ISSHL had significantly lower serum IGF-1 (*p* < 0.01) and HSP70 (*p* < 0.05) than the healthy control group. However, there was no significant difference in HSP70 between moderately severe ISSHL and healthy controls (*p* > 0.05). There was no significant difference in serum IGF-1 and HSP70 between moderately severe and severe ISSHL patients (*p* > 0.05). Profoundly deaf individuals had significantly lower serum IGF-1 (*p* < 0.01) and HSP70 (*p* < 0.05) levels compared to moderately severe individuals and significantly lower HSP70 levels compared to the severe group (*p* < 0.01, Figure 1).

### 3.4. Effects of HBOT on Serum IGF-1 and HSP70

**Figure 2:**
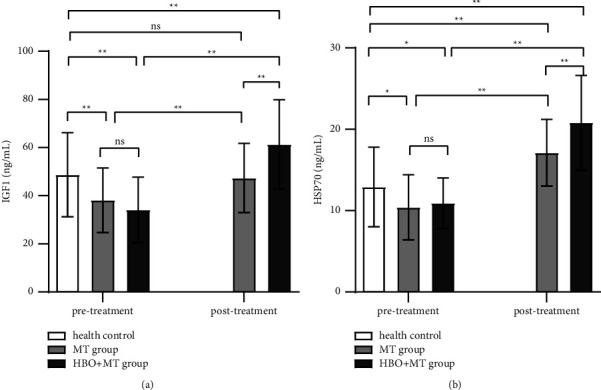
Effects of HBOT on the serum level of IGF-1 and HSP70. (a) IGF-1; (b) HSP70. *Note*: HBOT + MT group: HBOT and medical therapy group; MT group: medical therapy group. ^∗^*p* < 0.05. ^∗∗^*p* < 0.01. ns: *p* > 0.05.

Baseline serum IGF-1 and HSP70 levels were not different between the MT and HBOT + MT groups (both *p* > 0.05) but were lower in ISSHL compared to the healthy control group (*p* < 0.05 and *p* < 0.01, respectively; Figure 2).

Serum IGF-1 and HSP70 levels were increased after treatment in the HBOT + MT and MT groups (both *p* < 0.01), but they were significantly higher in the HBOT + MT group compared to the MT group (*p* < 0.05 and *p* < 0.01, respectively; Figure 2).

In addition, △IGF-1 and △HSP70 were greater in HBOT + MT than in the MT group (△IGF-1: 27.2 ± 8.9 ng/mL versus 9.3 ± 19.8 ng/mL; △HSP70: 9.9 ± 4.1 ng/mL versus 6.7 ± 3.6 ng/mL, *p* ≤ 0.001).

### 3.5. Correlations between △PTA, △IGF-1, and △HSP70

**Figure 3:**
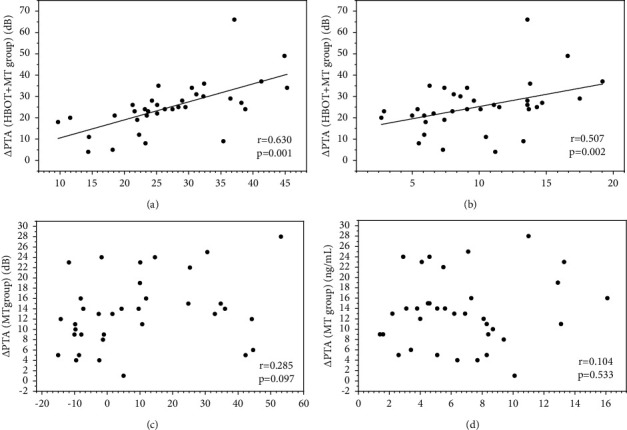
Correlations between △PTA and serum levels of △IGF1 or △HSP70 after treatment. Correlation between △PTA and serum level of (a) △IGF-1 in the HBOT + MT group, (b) △HSP70 in the HBOT + MT group, (c) of △IGF-1 in the MT group, and (d) △HSP70 in the MT group. *Note*: HBOT + MT group: HBOT and medical therapy group; MT group: medical therapy group.

**Figure 4:**
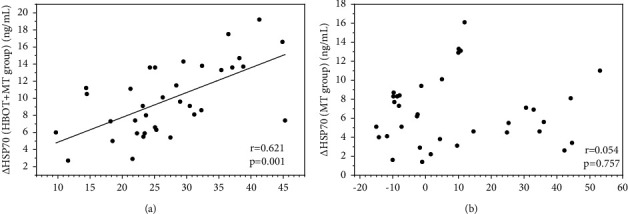
Correlation between the serum level of △IGF-1 and △HSP70 after treatment. Correlation between the serum level of △IGF-1 and △HSP70 in the (a) HBOT + MT group and (b) MT group. *Note*: HBOT + MT group: HBOT and medical therapy group; MT group: medical therapy group.

Correlation analysis showed a significant positive correlation between △IGF-1 and △HSP70 after HBOT and hearing gain in the HBOT + MT group. Specifically, greater hearing improvement was significantly associated with a larger increase in serum IGF-1 and HSP70 after HBOT (IGF-1: *r* = 0.630, *p*=0.001, Figure 3(a); HSP70: *r* = 0.507, *p*=0.002; Figure 3(b)). In addition, we observed a highly positive correlation between △HSP70 and △IGF-1 after treatment in the HBOT + MT group (*r* = 0.621, *p*=0.001) (Figure 4(a)). However, no such correlation was detected in the MT group (*p* > 0.05, Figures 3(c), 3(d), and 4(b)).

## 4. Discussion

Our study demonstrates the beneficial effects of HBOT in patients with ISSHL. HBOT increased serum levels of IGF-1 and HSP70; the magnitude of this change was positively associated with improved hearing. Furthermore, there was a correlation between the degree of change in IGF-1 and HSP70.

We enrolled only moderately severe to profound patients in this study for two reasons. First, all included participants were inpatients; mild to moderate cases are mainly treated in outpatient clinics. Second, we wanted to reduce the impact of spontaneous recovery on our results. In ISSHL, the spontaneous recovery rate ranges from 25% to 39%, with lower spontaneous recovery rates among patients with more severe cases [18]. We showed that combining HBOT and medical therapy improved recovery (80.0% versus 57.1%) and hearing gain (25.1 ± 11.9 dB versus 13.3 ± 6.8 dB), compared to medical therapy alone. Consistent with our findings, several systematic reviews have shown that adding HBOT to steroid therapies can benefit patients with severe to profound ISSHL [4, 19].

HBOT has been used to treat inner-ear disorders since the 1960s. In studies of acute noise damage, HBOT improved the anatomical pattern of damage detected by scanning electron microscopy and reduced the number of injured cochlear HCs detected by distortion product otoacoustic emission [20]. To date, the molecular mechanisms underlying the positive clinical effects of HBOT remain unclear. The basic strategy behind utilizing HBOT in ISSHL is to reverse the lack of oxygen in the inner ear, which is the final event triggering cochlear damage regardless of the trigger [21]. By using HBOT, it is possible to maximize the oxygen partial pressure supplied to the inner ear. Studies have found that patients with sensorineural hearing loss have decreased perilymphatic oxygen tension, but HBOT increases the oxygen tension in the perilymphatic fluid by 450% [22]. HBOT can minimize edema and ischemic damage after SSNHL, improve microcirculation, and promote angiogenesis [4, 22]. Furthermore, in our previous study, we provided evidence that HBOT reduces the expression of inflammatory cytokines in peripheral blood, thereby increasing the hearing level of ISSHL patients [23].

Studies conducted in recent years have supported the hypothesis that IGF-1 and HSP70 are essential for protecting HCs. Animal experiments and *in vitro* studies have shown that IGF-1 protects HCs against damage from noise exposure, ischemia, and ototoxic drugs [24]. A retrospective study of 25 patients found that topical application of exogenous IGF-1 aided the restoration of PTA levels in patients with systemic steroid-refractory ISSHL [25]. Another study showed that exosomal HSP70 protected HCs from death through a paracrine mechanism [10].

We showed that serum IGF-1 and HSP70 were decreased in moderately severe to profound unilateral ISSHL patients compared to healthy individuals. To the best of our knowledge, this is the first study to test serum IGF levels in ISSHL patients. We found that IGF-1 was significantly lower in profound than moderately severe patients, suggesting that IGF-1 levels may be related to the degree of hearing loss. In a study on IGF-1 haploinsufficiency, Celaya et al. [26] proposed that IGF-1 circulating levels have a threshold under which there is less effective cochlear control of the inflammatory response and survival signaling. As for serum HSP70, our results contrast with those of Park et al. [13], who found serum HSP70 to be significantly higher in patients with ISSHL. However, 41.8% of their cases had mild or moderate hearing loss, whereas we excluded such cases from our study. Furthermore, there were no profound cases in their study, compared with 14.3% in ours. In addition, we found that the degree of hearing loss affected the levels of serum HSP70; there was no difference in serum HSP70 between moderately severe cases and healthy controls, but serum HSP70 was lower in profound cases than in moderately severe and severe individuals. As such, the inclusion of cases with different severities of disease likely explains these diverse findings. In addition, both studies included relatively modest cohorts (*n* = 67 for Park et al. and *n* = 70 in the present study). Additional studies with larger cohorts of patients with ISSHL of various severities are needed to properly model how serum IGF-1 and HSP70 levels are associated with various disease parameters.

To explore the possible mechanisms of HBOT, we further investigated the effect of HBOT on serum IGF-1 and HSP70 levels and their relationship with hearing gain. To the best of our knowledge, this is the first study on changes in serum IGF-1 and HSP70 levels after HBOT in patients with sudden hearing loss. Studies on patients with diabetes-related foot problems have shown that HBOT can increase the level of IGF-1 in serum, improve diabetic microangiopathy, and promote wound healing [27]. Ueng et al. [28] studied articular cartilage injury *in vitro* and *in vivo* and found that HBOT can prevent nitric oxide-induced apoptosis by enhancing HSP70 expression. Differing from our results, Düzer et al. [29] reported decreased posttreatment serum anti-HSP 70 levels in recovered ISSHL patients. Anti-HSP 70 is thought to be raised against infectious agents and trigger autoimmune responses in human target organs [29]. However, the study by Düzer excluded patients with systemic diseases, while the proportions of patients with hypertension, diabetes, and hyperlipidemia in our study were 37.1%, 22.9%, and 35.7%, respectively. Therefore, we speculate that different etiologies may be partly responsible for these contrasting results: our study had more cases with vascular contributions to the disease. In comparison, Düzer had more cases arising from infections and other immune triggers. Our results suggest that HSP70 may be more likely to have a protective effect on patients with moderately severe to profound unilateral ISSHL caused by vascular factors.

In our previous study, we found that HBOT can improve the hearing level in ISSHL patients by attenuating TLR4/NF-*κ*B-mediated inflammation and reducing the expression of inflammatory cytokines in peripheral blood [23]. The work done by Celaya et al. [26] supports that IGF-1 plays a central role in maintaining cochlear homeostasis and regulating inflammation, which are implicated in sensorineural hearing loss. Some evidence suggests that IGF-1 inhibits TLR4 and NF-*κ*B signaling. Pinto–Benito et al. [30] found that IGF-1 treatment counteracted TLR4 expression in males. Based on *in vitro experiments*, IGF-1 might serve as a potential therapeutic target for cirrhosis and intestinal barrier dysfunction via its inactivation of the TLR4/MyD88/NF-*κ*B pathway [31]. HSP70 is also associated with reduced inflammation. In a mouse model, upregulation of HSP70 protein expression-alleviated spinal cord injury by inhibiting the NF-*κ*B signaling pathway to reduce lipopolysaccharide-induced expression of the inflammatory cytokine TNF-*α* [32]. Neuroinflammation is one important mechanism in Alzheimer's Disease, and HBOT has been reported to ameliorate the pathophysiology of Alzheimer's disease in a mouse model by attenuating neuroinflammation [33, 34].

The present study found that increased serum IGF-1 and HSP70 levels after HBOT are associated with improved hearing. Therefore, we surmise that HBOT improves the hearing of patients with ISSHL via the upregulation of IGF-1 and HSP70. We also speculate that the upregulation of IGF-1 and HSP70 can improve hearing by inhibiting inflammation and protecting HCs. Further studies involving animal models are required to verify these hypotheses.

Our study had two main limitations. First, the number of patients enrolled was relatively small (*n* = 35 for each group), precluding more complex analyses of variable relationships. Second, all patients were treated in the same HBOT center, thus limiting generalizability.

## 5. Conclusions

Administering HBOT in addition to medical therapy can improve the hearing of patients with moderately severe to profound unilateral ISSHL. The improvements are related to the upregulation of IGF-1 and HSP70.

## Figures and Tables

**Table 1:** Demographic data, comorbidities, and audiometric characteristics of the study cohort.

Clinical characteristics	ISSHL groups		Health control (*n* = 30)	*p* value
	HBOT + MT group (*n* = 35)	MT group (*n* = 35)		
*Sex* (%)				0.83
Male	15 (42.9%)	17 (48.6%)	15 (50%)	
Female	20 (67.1%)	18 (51.4%)	15 (50%)	

*Age* (years, (mean ± SD))	47.9 ± 15.9	43.5 ± 15.2	44.9 ± 10.3	0.24

*Comorbidities* (%)				
Hypertension	12 (34.3%)	14 (40.0%)		0.42
Type 2 diabetes	9 (25.7%)	7 (20%)		0.39
Hyperlipidemia	14 (40%)	11 (31.4%)		0.31

*Accompanying symptom* (%)				
Tinnitus	29 (82.9%)	26 (74.3%)		0.56
Dizzy	12 (34.3%)	16 (45.7%)		0.47

*Visit time* (days, (mean ± SD))	4.3 ± 2.0	3.9 ± 2.4		0.49

*Affected ear* (%)				
Left	19 (54.3%)	20 (57.1%)		0.80

Right	16 (45.7%)	15 (42.9%)	

*Degree of hearing loss* (%)				0.24
Moderate-severe (56–70 dB)	9 (25.7%)	13 (37.1%)		
Severe (71–90 dB)	22 (62.9%)	16 (45.7%)		
Profound (≥91 dB)	4 (11.4%)	6 (17.1%)		

HBOT + MT group: HBOT and medical therapy group; MT group: medical therapy group.

**Table 2:** Comparison of hearing improvement in ISSHL patients (dB, $\bar{x}$ ± *s*).

Groups	*n*	Initial PTA	After-treatment PTA	Hearing gain
HBOT + MT group	35	80.9 ± 11.2	55.8 ± 17.2^bc^	25.1 ± 11.9^d^
MT group	35	78.8 ± 13.8	65.5 ± 14.8^b^	13.3 ± 6.8

^a^Data are shown as mean ± SD. ^b^*p* ≤ 0.001 vs. initial PTA. ^c^*p* < 0.05 vs. MT group. ^d^*p* ≤ 0.001 vs. MT group. HBOT + MT group: HBOT and medical therapy group; MT group: medical therapy group. PTA: pure-tone average.

## Data Availability

The data used to support the findings of this study are available from the corresponding author upon request.
